# Book review of “Here, there, everywhere—A memoir” by Peter Almond

**DOI:** 10.1002/acm2.13825

**Published:** 2022-11-15

**Authors:** Bruce J. Gerbi

**Affiliations:** ^1^ Department of Radiation Oncology University of Minnesota Minneapolis Minnesota USA

The book “Here, there, everywhere—a memoir” is a collection of memories and experiences by Peter Almond, one of the most eminent medical physicists of the last 50 years. Peter started his medical physics career in 1959 in Bristol, England at the suggestion of his undergraduate physics advisor. This launched him on a path that eventually led to MD Anderson Hospital and Cancer Center in Houston, TX, and a career nearly unparalleled by others in medical physics. Peter went on to win the Coolidge Award of the American Association of Physicists in Medicine (AAPM), the highest honor given by the AAPM. He was also a Marvin MD Williams recipient and was chosen as a fellow of the American College of Medical Physics and the AAPM. Peter contributed greatly to the application of high‐energy electrons in radiation therapy and the calibration of high‐energy photon and electron beams for use in radiation therapy.

One of the most important publications of the AAPM, the report of Task Group 51 (TG‐51), was chaired by Dr. Almond and has been the standard for linear accelerator calibrations of photons and electrons for more than 20 years. It is the calibration standard used not only by the United States but also by Canada along with many other areas of the world. Peter was also instrumental in the writing of TG‐21, the calibration protocol that preceded TG‐51. Peter has led or contributed to numerous AAPM Task Group reports, publications in the historical literature, and other significant works in the field of medical physics. He is directly responsible not only for calibration accuracy but also for the improved outcomes of radiation therapy over the course of the last six decades.

This memoir stemmed from a gift from his granddaughter who had heard his many stories growing up and thought that grandpa should write them down. Her gift was to enroll him in an autobiography writing course at Rice University. The endpoint was this collection of stories consisting of 28 different chapters and approximately 143 pages. These stories fell into three distinct categories: “Here” that describes aspects of his life in the United States after leaving England; “There” describes his early life in England dealing with his early education and interactions with his brother and family, and “Everywhere” comprising stories of trips and experiences that happened during his professional life in many areas of the world.

The book is written in roughly chronological order but not strictly so. The first part of the book concentrates on Peter's early life in England. He was born in Downton, England in 1937 and his first awareness of the world began during the World War II. This was a time of the London bombings, buzz bombs flying erratically overhead, observation balloons, and the constant reminder of a world at war. His father, a Baptist minister, was intimately involved with the war effort and there were many families and soldiers that would need and benefit from his ministry. Peter often accompanied his father on visits to people's homes where he was exposed to the suffering of other people and the effects of abject poverty. The impact of these interactions would have a lasting effect.

Three chapters in this portion of the book, “Son of the Manse,” “Twins,” and “Late bloomers,” give some of the best insights into Peter's (and his brother's) formative years. Being the son of the minister keeps you constantly in the public eye and lets you know that you are being held to a higher standard. Sermons beginning with “I know a little boy who…” leaves little doubt as to who is providing the example of what not to do.

The chapter “Twins” starts with an instructive tone and describes exactly how Peter and his brother John were mirror twins, a 1‐in‐1000 phenomenon. Peter describes some of the usual things that all twins are forced to endure and it makes for enjoyable reading. The lighthearted tone changes abruptly mid chapter and caught me completely off guard. He tells of his brothers battle with pancreatic cancer and the events of the last bit of time that they spent together. It is a very intimate chapter. I have read it several times and have been moved each time.

“Late bloomers” was a complete surprise to me, a chapter in which Peter describes his early experiences in the English educational system. He tells how both he and his brother failed the elevens‐plus exam, the test that determines whether you will proceed to higher educational levels. They transferred to a private school in the hope of passing the thirteen‐plus exam, a second chance at a pathway to higher education. Every school day, the twins had to endure a 30‐min bike ride in the cold English winter to catch a train, followed by a long walk or bus ride to the school. The way home was a reverse of the previous one. This routine lasted for 2 years but in the end, both passed the thirteen‐plus exam. Their abilities blossomed from there finding them excelling in math, science, and sports. This led to a 1‐year fellowship in the United States in 1959 and a lifelong career in medical physics for Peter.

The second part of the book details Peter's life after his arrival in the United States. Inspired by his first year in medical physics at Bristol he contacted three centers in the United States and ultimately picked MD Anderson Cancer center for his future studies. Once in Houston he was not only able to quickly find a Baptist Church near his place of residence but also met his future wife. He was to attend this church for many years and keep close ties with the community throughout his life. This section of the book accentuates his lifelong faith and commitment that developed in his early years and life.

The final chapters of the book, the “Everywhere” portion, describe numerous trips to different parts of the world. As an expert in Medical Physics, he delivered many talks from India, to Japan, to Africa. He viewed these trips as opportunities to learn the cultures of other countries and saw them as opportunities to learn about the local people. We see the world through Peter's eyes and interactions with the common people of these countries.

The specific theme that binds together all stories and experiences in the book is Peter's faith and his expression of it in his daily life. The application of his faith and the general atmosphere surrounding faith in which Peter was raised influenced all activities of his life. We see this most in his work as a medical physicist, but this memoir reveals much more. It gives us the chance to know Peter not only as a fellow colleague but as a person. Through this work, I have come to know more about his personal life, childhood, aspirations, motivations, experiences, and faith than I do about most other people. I have been able to share his joys and sadness and his perspectives of the world. It is both thought provoking and an inspiration, and I highly recommend it. I only wish that it would have been longer.

